# Wilhelm His Sr. and the development of paraffin embedding

**DOI:** 10.1007/s00292-021-00947-4

**Published:** 2021-07-08

**Authors:** Tim van der Lem, Merijn de Bakker, Gerhard Keuck, Michael K. Richardson

**Affiliations:** 1 Institute of Biology, IBL, Sylvius Laboratorium, Leiden University, Sylviusweg 72, 2333BE Leiden, The Netherlands; 2 Geißspitzweg 8, 65929 Frankfurt, Germany

**Keywords:** Chick embryo, Histology, Microtome, Histopathology, Tissue embedding, Hühnerembryo, Histologie, Mikrotom, Histopathologie, Gewebeeinbettung

## Abstract

Paraffin histology is one of the most important and commonly-used laboratory techniques in diagnostic histopathology. The discovery of paraffin embedding is often attributed to the pathologist Edwin Klebs. Klebs was following the lead of Stricker, who embedded embryos in a mixture of hot stearin and white beeswax. We show that Klebs experimented with paraffin wax for embedding tumour tissue. But he quickly rejected it as unsuitable because paraffin wax did not infiltrate the tissue. One of Klebs’ correspondents, embryologist Wilhelm His, Sr., learned of Klebs’ experiments and decided to try paraffin embedding. His dehydrated chicken embryos in alcohol, cleared them in lavender oil, and dripped hot paraffin wax onto them. This process allowed His to cut good sections. Here, we have replicated His’s paraffin embedding protocol in order to determine whether His had indeed made the landmark discovery of infiltration embedding with paraffin wax. We followed the protocol that he gives in his 1868 monograph on the early development of the chicken. The protocol described by His failed, in our hands, to yield sections of the quality that he illustrates in his monograph. Typically, the tissue disintegrated when sectioned due to poor infiltration of the wax. Usable sections could only be obtained if His’s protocol was modified by melting the embedded embryos in fresh paraffin wax. One explanation for our findings is that we failed to faithfully replicate His’s protocol. Another is that his protocol was incomplete. We suggest that His is likely to have discovered and perfected infiltration embedding with paraffin wax but did not publish a complete protocol.

**Supplementary Information:**

The online version of this article (10.1007/s00292-021-00947-4) contains supplementary material, which is available to authorized users.

## Abstract (de)

Die paraffinbasierte histologische Untersuchung ist eines der wichtigsten angewendeten Laborverfahren in der diagnostischen Histopathologie. Die Entwicklung der Paraffineinbettung wird oft dem Pathologen Edwin Klebs zugeschrieben. Klebs orientierte sich an den Arbeiten von Stricker, der Embryonen in eine Mischung aus heißem Tristearin und weißem Bienenwachs einbettete, und experimentierte mit Paraffin, um Tumorgewebe einzubetten. Er verwarf den Ansatz aber rasch, da Paraffin das Gewebe nicht infiltrierte. Klebs stand im Briefwechsel mit dem Embryologen Wilhelm His Senior, der von dessen Experimenten erfuhr und beschloss, sich an der Paraffineinbettung zu versuchen. His entwässerte Hühnerembryonen in Alkohol, klärte sie mit Lavendelöl und träufelte heißes Paraffin darüber. Dieses Verfahren ermöglichte ihm die Anfertigung guter Schnitte. In der vorliegenden Arbeit haben wir His’ Protokoll der Paraffineinbettung nachvollzogen, um zu ermitteln, ob His tatsächlich die Infiltrationseinbettung mit Paraffin gelungen ist. Wir befolgten das Protokoll aus seiner 1868 erschienenen Monografie. Anhand der Anweisungen von His gelang es uns nicht, Schnitte mit der von ihm illustrierten Qualität herzustellen. In der Regel zerfiel das Gewebe beim Schneiden wegen schlechter Wachsinfiltration. Brauchbare Schnitte wurden nur bei Abwandlung von His’ Protokoll durch das Wiedereinschmelzen der eingebetteten Embryos in frischem Paraffin erreicht. Möglicherweise ist es uns nicht gelungen ist, das Protokoll von His detailgetreu zu replizieren, oder das Protokoll war unvollständig. Nach unserer Einschätzung hat His wahrscheinlich die Infiltrationseinbettung mit Paraffin erfunden und perfektioniert, aber kein vollständiges Protokoll veröffentlicht.

## Introduction

Embedding is a technique used to prepare tissues for microscopic analysis. It entails the placing of the specimen in a solid mass while it is sectioned using a microtome [22]. The mass should be hard enough to support the tissue, but soft enough to be cut easily into sections. There are two types of embedding [7]: peripheral embedding simply encases the tissue, supporting it only on the outside. By contrast, infiltration or interstitial embedding supports the tissue outside and inside, because the embedding mass completely permeates the tissue. Infiltration may require an intermediate reagent: a solvent that is miscible both with the alcohol used to dehydrate the tissue, and with the embedding medium [7, 21, 22]. Many intermediate reagents also act as clearing reagents, rendering the tissue optically transparent [22, p. 68].

One widely-used embedding medium is paraffin wax (often simply called ‘paraffin’). Paraffin wax is a petroleum derivative consisting of a mixture of straight and branched hydrocarbons [37]. It is poorly soluble in alcohol [30, p. 356], and for this reason, can only be used for infiltration embedding in combination with an intermediate reagent. One of the many useful properties of paraffin wax is that thin sections (5–7 µm) tend to adhere to one another in a ribbon as they are cut, allowing several sections to be mounted on the slide in one operation.

Paraffin-based histology is so widely used today that it is often referred to as ‘routine’ histology [3, 26, 27]. It is used in diagnostic histopathology to study abnormal cell and tissue structures [32]. It is also used in many areas of biomedical research to study tissue structure [26] and gene expression patterns [24].

### Histotechnique up to the 1860s

By the early 1860s, botanists had long been able to make histological sections. Fresh plant tissues are often sufficiently rigid to be sectioned by hand using a razor [36]. Microtomes were also available. In 1770, Hill described a microtome or ‘cutting engine’ designed by Cummings for the sectioning of woody tissue [12]. To hold the specimen during sectioning, botanists would often clamp it between strips of a soft, supporting material such as the pith from young branches of the elder tree (*Sambucus nigra*) [5].

In contrast to plant tissues, fresh animal and human tissues are typically too soft to be cut into fine sections; they therefore need to be hardened or embedded. Usually, soft animal tissues were hardened with alcohol or a fixative [29 p. 460–473], or they were left outdoors in winter to freeze [38]. Adequate embedding techniques were not yet available for animal tissues. However, botanists were beginning to experiment with embedding media.

According to one anecdotal report, Eduard Fenzl had ‘years ago’ embedded small pieces of plant tissue in stearin in order to prepare them for sectioning [17, p. 11]. Apáthy reports a belief among some botanists that Fenzl also introduced paraffin wax as an embedding medium [2 p. 80–81 footnote 3], although this report is anecdotal. Schatz recommended injecting dry, friable wood specimens with molten stearin in order to render them suitable for sectioning [35 p. 66]. Stearin is a triglyceride of stearic acid [41], and at that time it was manufactured as an impure preparation of animal fats [33, p. 52].

### 1864: Salomon Stricker and infiltration embedding with beeswax and stearin

Stricker, in his studies of frog development (*Bufo* sp.), found that the tissue was too opaque to be studied adequately under the microscope [39]. He therefore decided to make microscopic sections. He fixed the embryos and larvae with chromic acid, then dehydrated and cleared them in absolute alcohol and turpentine. This treatment rendered the tissue transparent [39 p. 53] (and the turpentine presumably acted as an intermediate reagent). He then dripped a molten mixture of white wax and stearin onto the cleared embryos. The ‘white wax’ referred to by Stricker is likely beeswax bleached by exposure to the sun [30 p. 603]. He then sectioned the embryos (see his Plate I, [39]).

### 1867: Edwin Klebs and peripheral embedding with paraffin wax

Paraffin wax was introduced as an embedding medium by Edwin Klebs [9, 30, 40]. Klebs, professor of pathology at the University of Bern, was researching laryngeal tumours [18]. He noted that histopathology, and its application to the understanding and diagnosis of cancer, was still a poorly developed science. He made microscopic sections of the tumours using what he calls the ‘melting-down technique’ (*Einschmelzungs-Methode*). He recalls that this technique was probably first used in embryological research by Stricker [18 p. 207–208 n]. He later revised his recollection [19 p. 164] and attributed the invention of ‘melting-down’ to Rudolf Heidenhain, who used a concentrated gum Arabic solution as the medium. Later still, Klebs said that Heidenhain had since written to him denying that he was the originator of the technique [20, p. 206 n].

Klebs substituted paraffin wax for the beeswax and stearin used by Stricker and thereby introduced paraffin wax to histology. He dripped molten paraffin wax onto the tissue, which had been prepared either with or without alcohol [18 p. 207–208 n.]. He found the sections to be better than those cut from fresh tissue [18, p. 215].

In a later paper [19], Klebs said that he had been using paraffin for 5 years, and that other researchers, including Wilhelm His Sr., had also found it to be useful (p. 164). However, he complains that the wax does not adhere completely to the tissue and that gaps are thereby left. These gaps are a nuisance since they allow the tissue to move when sectioned (p. 165). The poor miscibility of paraffin wax with alcohol is a likely explanation for these problems. Whatever the case may be, Klebs abandoned paraffin wax in favour of a mixture of glycerine and isinglass (fish glue), which does penetrate the tissue [19, p. 165].

Curiously, Klebs’ 1869 publication is often cited as marking the introduction by Klebs of paraffin wax for histology [6, 40], when in fact it marks his abandonment of paraffin. That misunderstanding may have started with Long in his 1928 book *A History of Pathology* (reprinted as [25]).

### Wilhelm His Sr. (1868): infiltration embedding with paraffin wax

**Fig. 1:**
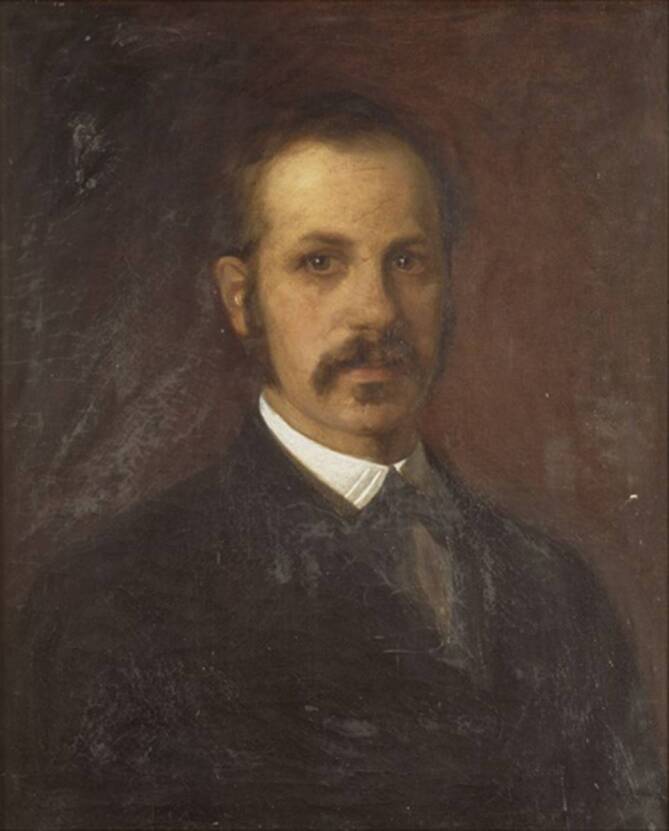
Portrait of Wilhelm His Sr. Painting (oil on canvas), 62 × 49 cm, signed Albert Winther, dated 189? (art collection of the University of Leipzig, inventory No. 1951:004, photographed by Karin Kranich; image rights: Kustodie der Universität Leipzig. The date is difficult to read and it is possible that this portrait shows a young middle-aged His from the 1870s or 1880s)

Wilhelm His Sr. (1831–1904) was an embryologist and professor of anatomy and physiology at Basel University and later at Leipzig University (Fig. 1). He published numerous important studies in the fields of pathology, anatomy and embryology [8]. His son, Wilhelm His Jr. (1863–1934), discovered the atrioventricular bundle (of His) [1].

His Sr. found it impossible to make good-quality sections using the techniques of the time [15]. He became aware of Klebs’ paraffin wax, and saw that it held promise [15, p. 181]. His modified Klebs’ technique by including dehydration with alcohol and clearing in lavender oil or Canada balsam. Canada balsam is an oleoresin from the fir *Abies balsamea*; lavender oil is distilled from *Lavandula* sp., often *L. angustifolia*.

Wilhelm His persisted with paraffin wax where Klebs had left off, a fact noted by Klebs himself [19, p. 164]. His described paraffin wax as a ‘wonderful substance’ that he had learned about from Klebs [15, p. 181]. The use of lavender oil by His is intriguing since it may have functioned as an intermediate reagent, enabling the wax to infiltrate the tissue. His may therefore have discovered, perhaps serendipitously, the process of infiltration embedding using paraffin wax.

**Fig. 2:**
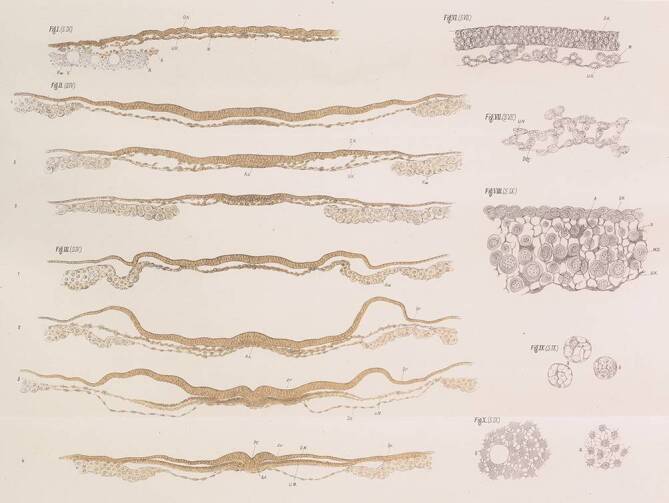
Histological sections illustrated by His in his monograph on the chicken embryo. Plate VI from Ref. [15]. Note that the quality of the sections is good, and cellular detail is shown, suggesting that the tissue was properly infiltrated with paraffin wax. Transverse sections of chicken embryos at his stages I–IV (formation of the endoderm to formation of first somite)

His described his protocol for embedding chicken embryos in his monograph on the chicken embryo [15, p. 180–182] (reproduced here in the Supplementary Information). This yielded what appear to be good quality sections (Fig. 2). He dehydrated embryos through a graded alcohol series then soaked them in lavender oil as a clearing agent to render them transparent for study as wholemounts. He sometimes mounted an embryo in Canada balsam and closed it with glass covers in a chamber slide so that he could examine it from both sides (dehydration and clearing were already established techniques [43, p. 12]).

Embryos cleared in lavender oil were then embedded. His placed the embryos on a gutta-percha plate and dripped molten paraffin wax onto them. After sectioning using his own microtome [13], he mounted the sections on glass slides and removed the paraffin using chloroform or benzine [15, p. 181].

Hensen recalls that paraffin was endorsed by His [11]. Apáthy refers to ‘the paraffin method of His’ [2]. Only 1 year after His’s publication, Dr. Moritz Roth in Greifswald was using the ‘method given by His for embryos’ [34 p. 246]. Waldeyer attributes paraffin embedding to Klebs and His [42].

The way paraffin embedding is performed today by pathologists and researchers is remarkably similar to His’s technique in terms of the basic steps. The main improvements regard the choice of intermediate reagent, the formulation of the paraffin mixture and the sectioning procedure, for which automatic microtomes are nowadays used, routinely yielding 5‑ to 7‑μm sections. For details on the improvement of paraffin embedding over the years, see [22, 31].

### His’s protocol and our testing of it

Judging from the quality of the sections illustrated in His’s monograph, he presumably achieved infiltration with paraffin. The issue addressed in this article is whether the protocol that he published [15] was indeed capable of achieving infiltration.

We attempted to faithfully replicate His’s protocol. His reports that he dripped hot wax onto the tissues while they were mounted on a gutta-percha plate. However, he provides few details. We presume that he used gutta-percha because its low thermal conductivity [28] meant that the wax could remain molten for some time before cooling and solidifying. Since we were unable to obtain a plate of gutta-percha, we used plates made of Bakelite or cork, both of which have low thermal conductivity [10, 23]. Unable to produce useable sections with His’s protocol, we tried several variations on that protocol (summarised in Table 1), including an additional step of melting the paraffin-embedded embryos down in fresh molten paraffin wax. In all cases, we sectioned at 50 µm because this is the standard thickness used by His for sectioning chicken embryos [14, p. 383].

**Table 1:** Summary of protocols and results

*N*	Fix	Dehydration protocol	50:50	Int. reagent	Blot	Base	Drying	Drip	Re-embedding	Result
5	OsO4	50% (1 h), 70% (1 h), 2 × 100% (1 h each)	No	Lavender	No	Bake	No	Yes	No	_a_
1	OsO4	50% (1 h), 70% (1 h), 2 × 100% (1 h each)	Yes	Lavender	No	Bake	No	No	No	_b_
1	OsO4	50% (1 h), 70% (1 h), 2 × 100% (1 h each)	No	Lavender	Yes	Bake	No	Yes	No	_a_
1	OsO4	50% (1 h), 70% (1 h), 2 × 100% (1 h each)	No	Lavender	Yes	Bake	No	Yes	Yes	_d_
1	OsO4	50% (1 h), 70% (1 h), 2 × 100% (1 h each)	No	Lavender	Yes	Bake	No	Yes	No	_a_
1	OsO4	50% (1 h), 70% (1 h), 2 × 100% (1 h each)	No	Lavender	Yes	Bake	30 min	Yes	No	_a_
1	OsO4	50% (1 h), 70% (1 h), 2 × 100% (1 h each)	No	Lavender	Yes	Bake	1 h	Yes	No	_a_
2	OsO4	50% (1 h), 70% (1 h), 2 × 100% (1 h each)	No	Lavender	Yes	Cork	No	Yes	No	_a_
1	OsO4	50% (1 h), 70% (1 h), 2 × 100% (1 h each)	No	Lavender	Yes	Cork	30 min	Yes	No	_a_
1	OsO4	50% (1 h), 70% (1 h), 2 × 100% (1 h each)	No	Lavender	Yes	Cork	1 h	Yes	No	_a_
2	OsO4	50% (1 h), 70% (1 h), 2 × 100% (1 h each)	No	Lavender	Yes	Cork	No	Yes	Yes	_d_
2	OsO4	50% (1 h), 70% (1 h), 2 × 100% (1 h each)	No	Lavender	Yes	Bake	No	Yes	No	_a_
2	OsO4	50% (1 h), 70% (1 h), 2 × 100% (1 h each)	No	Lavender	Yes	Bake	No	Yes	Yes	_e_
2	OsO4	50% (1 h), 70% (1 h), 2 × 100% (1 h each)	Yes	Lavender	No	Plastic	No	No	n.a.	_b/c_
6	Bouin	50% (1 h), 70% (1 h), 95% (1 h), 3 × 100% (1 h each)	Yes	Histoclear	No	n.a.	No	No	No	_e_

*N* number of embryos, *Fix* fixative, *50:50* mixture of equal proportions of intermediate reagent and paraffin wax before embedding in pure paraffin wax, *Int. reagent* intermediate reagent, *Blot* blotting of lavender oil before applying paraffin, *Drip* dripping of paraffin on embryo, *Bake* Bakelite, *Lavender* lavender oil, *Bouin* Bouin’s fluid

Under ‘Results’, superscripted letters ^a–e^ are a subjective indication of the quality of the sections where ^a^ = poor quality, tissue badly torn, ^e^ = excellent quality, no tears

## Materials and methods

### Ethics statement

All animal experimental procedures were conducted in accordance with local and international regulations. The local regulation is the *Wet op de dierproeven* (Article 9) of Dutch Law (National) and the same law administered by the Bureau of Animal Experiment Licensing, Leiden University (Local). This local regulation serves as the implementation of *Guidelines on the Protection of Experimental Animals *(Council of Europe, Directive 86/609/EEC), which allows chicken embryos to be used before the moment of hatching (approximately 21 days of incubation at 38 °C). Because embryos to be used here were no older than 3 days of incubation, no license was required by Council of Europe (1986), Directive 86/609/EEC, or the Leiden University ethics committee.

### Embryos

Fertilised eggs of the White Leghorn chicken (*Gallus gallus*) were provided by a commercial supplier (Drost Loosdrecht B.V, Loosdrecht, the Netherlands). We incubated the eggs for 2.5 days at 38 °C in a humidified incubator with stationary shelves. Embryos were staged according to Hamburger and Hamilton and removed from the eggs into phosphate-buffered saline (PBS).

### Fixation, embedding and sectioning

**Fig. 3:**
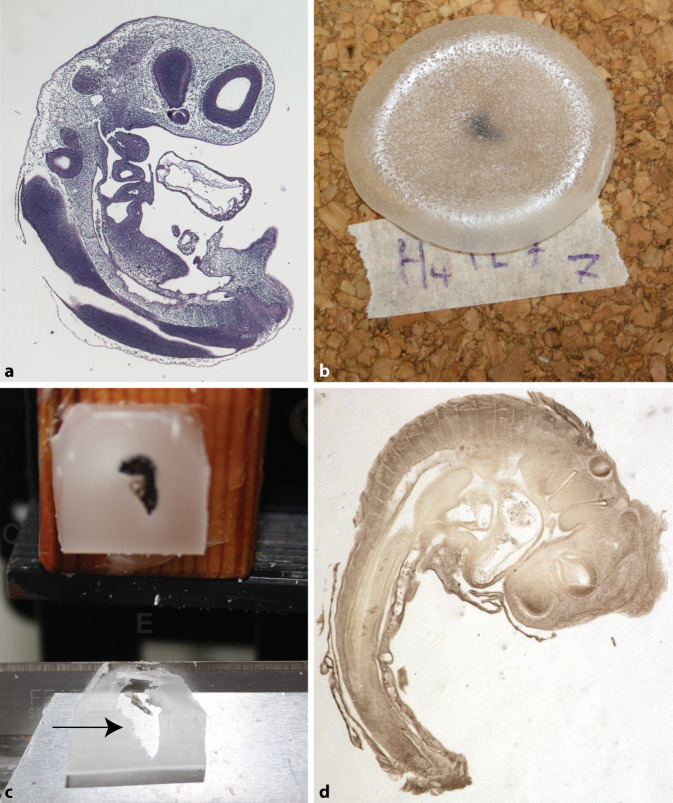
Histological sections from this study. **a** Control section of chicken embryo using modern, routine histology [4, 26, 32] (7 µm, haematoxylin and eosin stain). **b** Chicken embryo on cork plate after being dripped with paraffin wax according to His’s protocol. **c** Chicken embryo processed according to His’s protocol and an attempt made at sectioning (50 µm). Note that the tissue is not infiltrated; it is crushed and falls out, leaving a hole (*arrow*) in the paraffin when sections are cut. **d** A chicken embryo processed using His’s protocol but re-embedded in molten paraffin. Infiltration is now sufficient to allow good sections to be cut (50 µm, the brown stain is from the fixative osmium tetroxide)

As positive controls, we first used conventional histological processing techniques [4, 26, 32] to produce sections of 2.5-day chicken embryos at 7 µm stained with haematoxylin and eosin (Fig. 3a). We then attempted to prepare sections of 2.5-day chicken embryos using the protocol described by His (Supplementary Note 1). Because that protocol lacked details, we implemented a number of variations (listed in Table 1). In brief, embryos were fixed by dripping 0.5% osmium tetroxide solution onto them until they started turning brown (30–60 s). They were then dehydrated in graded ethanols to 100% and cleared overnight with an intermediate reagent, either lavender oil (‘*Lavendula officinalis*’; www.berivita.com) or Histo-Clear™ (National Diagnostics, Atlanta, USA).

We embedded several of the embryos in paraffin wax (Paraclear; Klinipath, Duiven, NL) at 62 °C by dripping molten paraffin wax onto them (Fig. 3b), as described by His. To do this, embryos were taken out of the lavender oil, placed on a plate of Bakelite or cork with approximately 20 µl of lavender oil adhering to them, and approximately 2.5 g molten paraffin wax (62 °C) was then dripped onto them. In some cases, the surplus lavender oil was first blotted away with filter paper or allowed to dry, either until the excess oil had evaporated, or until it was completely dry (Table 1). Other variations were as follows: Some embryos were taken from lavender oil to a 50:50 mixture of lavender oil and paraffin wax (62 °C, 1 h), then embedded in molten paraffin wax. Others, after having being processed according to His’s protocol, and having had paraffin wax dripped on them and allowed to cool, were melted down in fresh molten paraffin and embedded.

In all cases, the paraffin-embedded embryos were allowed to further solidify overnight, removed from the plate with a razor blade and mounted on 3‑cm^3^ pine wood blocks for sectioning at 50 µm. Sections were dewaxed and stained with haematoxylin and eosin and coverslipped with Eukitt mounting medium (Sigma-Aldrich, now Merck KGaA, Darmstadt, Germany).

## Results

We used cork or Bakelite bases (gutta-percha could not be obtained). On these substrata, the wax remained molten for a considerable period of time (25 min and 7 min, respectively), but we still did not obtain usable sections (Fig. 3c). When molten paraffin was dripped onto embryos with approximately 20 µL lavender oil adhering, the paraffin failed to penetrate the tissue. The sections disintegrated when mounted on the slides, probably because of the persistence of lavender oil in the embedding preparation. Only a few shreds of remaining tissue were adequately sectioned. Blotting away the excess lavender oil achieved a modest improvement in some sections, but still did not yield acceptable sections. We speculated that the paraffin was not molten long enough for it to mix with the lavender oil and infiltrate the tissue. To test this hypothesis, we tried adding an additional step to His’s protocol: re-melting the specimen (that had been dripped in paraffin wax) and then placing it in molten paraffin in the oven at 62 °C. This produced a dramatic improvement in the infiltration of the paraffin wax, and a corresponding improvement in section quality (Fig. 3c). An alternative addition to His’s protocol was also tried: an infiltration step in a 50:50 mix of lavender oil and paraffin wax. This produced some improvement in section quality, but not as great as with the re-melting and prolonged infiltration in molten paraffin wax.

## Discussion

More than before, the one who publishes a study is now being told that he also demonstrates the methods of investigation used. I will try to satisfy this demand in the following lines. (Wilhelm His Sr. [15]).

It seems unlikely to us that the protocol described by His in his 1868 mongraph [15] can produce sections of any quality. Certainly not the excellent sections he illustrates in that particular work (Fig. 2), nor the unbroken series of high quality sections that he must have needed to make his wonderful three-dimensional models of embryos [16]. When we followed his protocol faithfully, the tissue was poorly infiltrated and the sections mostly torn and unusable. Only when we added an infiltration step with molten paraffin to His’s protocol were the sections of acceptable quality.

It is possible that we failed to adequately replicate His’s protocol, although we tried several variations on his technique without success. Even when we allowed the embryos to remain in hot wax for 25 min (by dripping molten paraffin onto them while they were resting on a cork plate), infiltration by paraffin was still not achieved. It is possible that the paraffin wax used by us had different properties from that used by His. We used Paraplast, which consists of paraffin wax with some plastic polymers added (the manufacturer, Sigma Merk, told us that the exact composition is proprietary information).

This raises the possibility that His used a more prolonged infiltration step, but did not mention this in his protocol. Another possibility is that His used wax at a much higher temperature than the 62 °C used in routine histology today (and used here). In principle, very hot wax could have remained molten for longer and infiltrated faster. Unfortunately, His does not state the temperature of the wax he used.

The incompleteness of His’s protocol could have been an innocent oversight. If not, then it is possible that he did not want scientific competitors to copy his technique. It is also worth noting that sectioning was the basis of his commercially successful models. Whatever the case may be, we certainly do not intend to accuse such a great scientist of any sleight of hand. Rather, we suggest here that His should be credited with the landmark invention of infiltration embedding with paraffin wax. It is unfortunate that His did not publish a complete protocol, because that would have recorded his important innovation for posterity.

## Supplementary Information

English translation of His’s histology protocol. From [15, p. 180–182], with original footnotes

## Declarations

For this article no studies with human participants were performed by any of the authors. All applicable international, national, and/or institutional guidelines for the care and use of animals were followed (see section Material and Methods: Ethics statement). All studies cited were in accordance with the ethical standards indicated in each case.

The supplement containing this article is not sponsored by industry.
