# Supplementary material for: Wilhelm His Sr. and the development of paraffin embedding
Source: Pathologe. 2021 Jul 8;42(Suppl 1):55–61. doi: 10.1007/s00292-021-00947-4 (PMC8571243; doi:10.1007/s00292-021-00947-4)
Supplement: Supplementary file 1 — English translation of His’s histology protocol. From [15, p. 180–182], with original footnotes [file 292_2021_947_MOESM1_ESM.pdf]

ESM –Material zu "Wilhelm His Sr. and the Development of Paraffin Embedding

Supplementary Note 1

English translation of His's histology protocol. From [15] p. 180-182, with original footnotes.

III. Research Methods.

The handling of very young embryos is something that one has to learn the hard way. However the admirable studies of the founders of embryology (C. Fr. Wolff, Döllinger and Pander, v. Baer, Rathke and others) show how far one can go with a skilful hand, patience and the simplest of tools. Those workers made new discoveries that are difficult to equal, even with far more complicated techniques.

Some notes on the techniques used by the Würzburg school can be found in the Latin version of the dissertation of Pander. Those notes describe how Pander and his associates opened the eggs under water, a technique that originated with Dollinger, was later recommended by Erdl, and was recently adopted by Moleschott and Dursy. Some maceration in water, and pulling apart with needles, are the only other procedures that appear to have been applied by the older observers.

Now, more than ever, the authors of research papers are required to describe the methods that they used. I will try to satisfy this requirement here, and while the methods of egg-manipulation I have used have served me well, there are certainly other methods that may be better suited for some purposes.

Following the Würzburg method recently communicated by von Baer, I cracked the blunt end of the egg, broke the egg open, and then poured the contents into a shallow glass dish[1]. The chalazae are cut close to the yolk, either before or after decanting the egg contents, so that the innermost, viscous layer of albumen can be stripped from the vitelline membrane as a coherent sleeve. The embryo is caused to lie uppermost by rotating the yolk, the cutting

---

[1] This method of opening the egg is far simpler and safer than breaking the egg from above, as Moleschott and Dursy once again recommend.

round it in a circle, and lifting clear of the rest of the yolk by means of a large glass coverslip. This operation fails only if the albumen was not properly removed from the vitelline membrane. If this is done with the necessary care, 2 day embryos can easily be lifted off cleanly and brought under the microscope. In the case of younger embryos, however, a certain amount of yolk comes with the detached specimen. In the case of un-incubated eggs, the entire floor of the germinal cavity, together with some yellow yolk, also comes loose.

The removal of adherent yolk from the isloated embryo, and removal of the vitelline membrane, is carried out using iodine serum [animal serum with iodine as a preservative], which I pipette in a very gentle stream over the specimen. In this and the following operations until dissection, the coverslip is kept [in place] as both a support and a suitable means of transport of the preparation, which under these circumstances suffers little distortion or folding[2].

I now place the cleaned embryo, still adhering to the coverslip, under the microscope. At low and medium magnifications, it is easy to observe it from the dorsal side, and from the ventral side if one uses the humidity chamber of Kühne, which consists of a slide with an opening drilled through it, and the opening closed at the bottom with a plate. Of course, the [cavity thus formed] must be large enough to accommodate the embryo without the embryo touching the plate when it is turned upside down.

After I have completed my preliminary examination of the fresh embryo, I add a few drops of 0.5% osmium tetroxide until the embryo becomes slightly brown in colour. The osmium tetroxide is then removed by rinsing the embryo with dilute alcohol. Then it is placed, sequentially, in dilute, stronger and finally in absolute alcohol, and after it is sufficiently dehydrated, in essential oil (lavender oil)[3].

---

[2] Remak, to the best of my knowledge, was the first to use the method of extracting the embryo on a glass plate; he used fresh crayfish blood for rinsing, and I observed him doing this [with excellent results]. Erdl recommends warm salt water, also Moleschott and Dursy in their newest study, find c. 1% salt water to be optimal. The excellent medium of Max Schultze [animal serum with iodine as a preservative] combines the expediency of the former with the convenience of the latter method.

[3] On the whole, I did not vary this hardening method much because it served my goals very well. However, for special questions, and for more precise histological studies, other hardening methods such as chromic acid or gold preparations prove even more suitable; thus I made preparations with gold chloride which were superior to osmium preparations in their sharper definition of cell boundaries, but which otherwise lacked the finesse of osmium preparations.

When it has been prepared in this way, the embryo can be sealed between two coverslips in Canada Balsam, and cemented onto a microscope slide with a hole drilled in it. This mounting technique has the advantage that the preparation can be viewed from both sides at high magnification. — If the embryo is to be sectioned, I first bring it under the microscope again and make a drawing of it using a camera lucida at low magnification (Hartnak, System I or II) then embed it by dripping paraffin onto it on a small gutta-percha plate. I first got to know about the use of this excellent material [paraffin] for microscopic purposes from Klebs. The sectioning of the embryo is by means of a special apparatus [microtome] to be described elsewhere. —The cutting device [microtome] works as follows: the object is pushed, by means of a micrometre screw, under a vertically positioned steel plate, against which a razor blade, ground flat on one side [can be slid]. I got the idea of making this device from Hensen's cross-cutter. In my opinion, [my device] makes it easier to serial-section the embryo in the desired section plane and thickness without losing sections. The sections are placed one by one on the microscope slide, freed of any adherent paraffin [by rinsing them with] chloroform or benzine, and cemented in Canada Balsam. The numbered sections can, of course, be accurately compared not only with each other but also with the drawing of the intact embryo made earlier.

The whole operation from the opening of the egg to the cutting of sections is completed in 12-24 hours. As a rule, I section the embryos the day after I removed them from the egg, and [the results are less satisfactory] when things have been more protracted. Prolonged soaking in alcohol or essential oil leads to shrinkage of the embryo which is not seen when the processing was more rapid.

I attach just as much importance to the production of as complete a series of sections as possible, as I do to making an accurate drawing of the intact embryo. With young embryos, hardly any of which are alike, a secure understanding can only be achieved by comparing all relations of one and the same object as completely as possible; with older embryos of 2, 3, or 4 days old, we could become hopelessly confused if we do not know the exact position of the section and its relations to the previous and subsequent sections.

The principle underlying this research is to look at an embryo in surface view, and then examine it in longitudinal and transverse sections, like the different elevations of an architectural plan. This goal can only be reached indirectly; nonetheless an indirect approach

is adequate for the study of older embryos (from 2 to 4 days). If one has the surface view, and the corresponding series of cross-sections drawn with the camera lucida, and one knows exactly the position of each cross-section relative to the body axis, then one can draw the longitudinal axis of the dorsum with approximately right curvature; and using dividers, the correct position of the neural tube, aorta and son forth can be determined by measuring their depth on the cross sections. One thus obtains a sagittal section which, for older embryos, differs from the true section in that all points on the curved median surface come to lie in one plane, as if the embryo had been straightened out so that its median surface is flattened out. Of course one can also reconstruct coronal sections or sections in any other plane.

With the aid of the various sections of the same specimen, physical models can now be made. — At an earlier stage of my investigation, I made a number of models out of flexible materials such as lead sheets and leather and learned much from their construction. The models powerfully illustrate the folding principle [i.e. the principle that folding is an important mechanism of morphogenesis], more so than does the modelling of serial sections. However, the flexible models are of little value when complex folds have to be made. I would probably have stopped there if Freiburg artist Dr. A. [Adolf] Ziegler had not been there to help me. Through him, I became competent in the handling of modelling clay and wax. We produced, in collaboration, a series of models using the specimens and sections, the aim of which was to achieve the most accurate and naturalistic representation of the embryo.

Those who have never tried plastic modelling can hardly imagine the [level of precision and control] that it provides. Every detail, every apparent irregularity of a section takes on its own special meaning, every flaw [in the section] produces an error in the plastic model, and the detailed working through of such models with compasses and rulers gives a certainty of intuition that can scarcely be achieved in any other way.

____
